# Supplementary material for: The Tetraspanin CD9 Facilitates SARS-CoV-2 Infection and Brings Together Different Host Proteins Involved in SARS-CoV-2 Attachment and Entry into Host Cells
Source: Viruses. 2025 Aug 20;17(8):1141. doi: 10.3390/v17081141 (PMC12390632; doi:10.3390/v17081141)
Supplement: Supplementary file 1 [file viruses-17-01141-s001.zip › viruses-3762378-supplementary.pdf]

SUPPLEMENTARY FIGURE S1

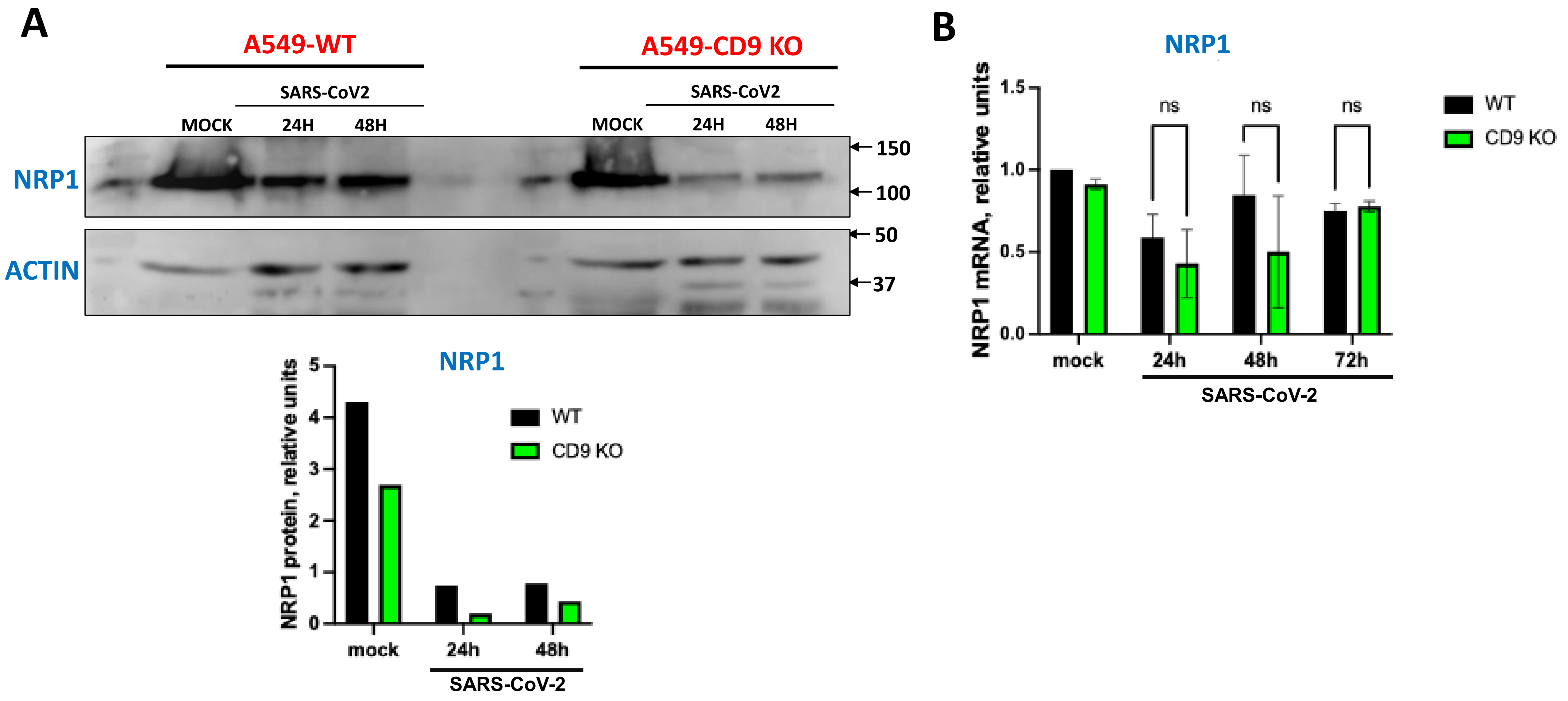

Supplementary Figure S1. CD9 expression affects NRP1 expression levels. A549-ACE2 WT and CD9 KO cells were left mock-infected or infected during 24, and 48 hpi. (A) Protein extracts were obtained and the levels of NRP1 and actin, as control, were analyzed by Western blot using specific antibodies. Molecular weights (in kilodaltons) are indicated on the right. Western blots were quantified by densitometry using ImageJ software, and normalized to the levels of actin in each sample (graphs on the bottom). (B) Total RNAs from mock-infected or SARS-CoV-2-infected cells were purified and the expression level of NRP1 mRNA was quantified by reverse transcriptase reaction followed by qPCR, and quantified to the levels of actin mRNA. Error bars represent standard deviations (SD) of results of measurements performed in triplicate wells.

SUPPLEMENTARY FIGURE S2

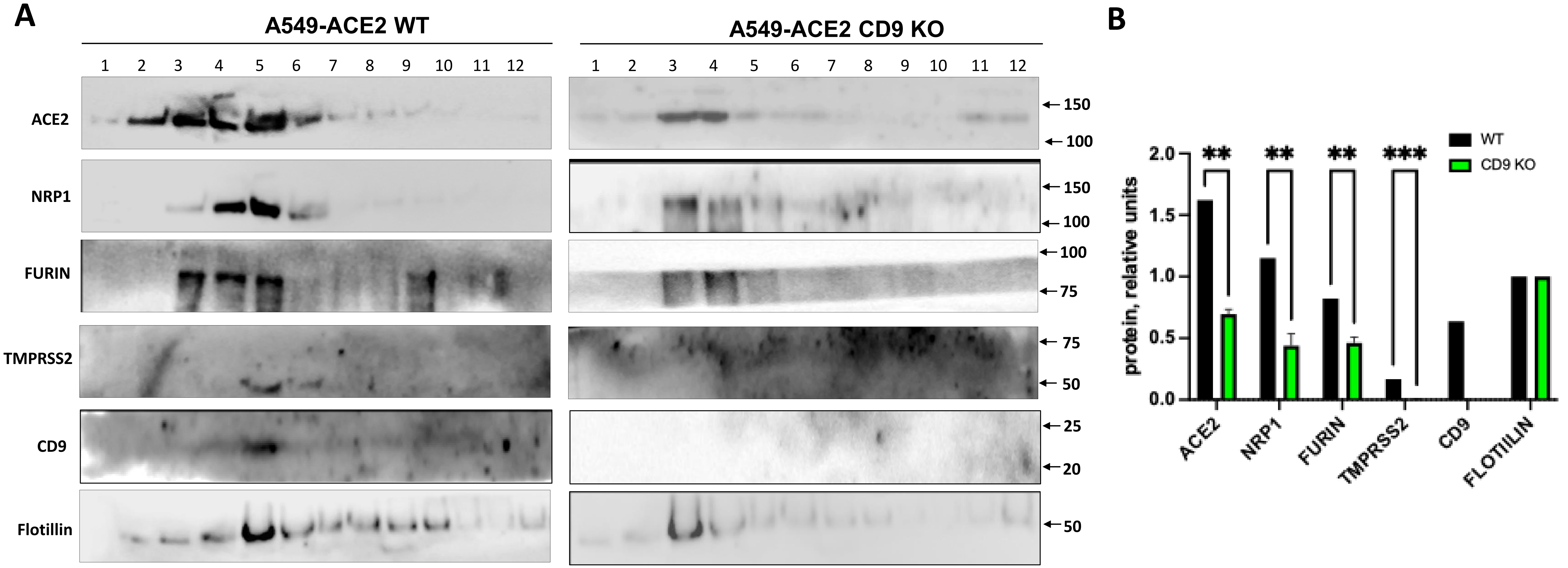

Supplementary Figure S2. CD9 localizes to the same transmembrane domains as ACE2, NRP1, furin and TMPRSS2, and increases the expression levels of these proteins. To analyze whether CD9, ACE-2, NRP1, furin and TMPRSS2 are located to the same membrane microdomains, a fractionation experiment using the Brij98 detergent was performed. Lysates from A549-ACE2 WT and CD9 KO cells were layered beneath a discontinuous sucrose step gradient, from which 12 fractions were collected from top to bottom after overnight ultracentrifugation. (A) Western blot analysis, using antibodies specific for ACE-2, NRP1, furin, TMPRSS2, CD9, and flotillin, as control, were performed using fractions containing the same amount of protein. The molecular weights are indicated on the right (in kilodaltons). The fraction numbers are indicated at the top. Two experiments were performed showing similar results, representative blots from the two experiments are shown. (B) The fraction 5 of the Western blots in WT cells, and the fraction 3 of the Western blots in fraction 3, showing the highest flotillin protein amounts, respectively, were quantified by densitometry using ImageJ software. The amounts of ACE2, NRP1, furin, TMPRSS2, and CD9 were normalised by the amount of flotillin in fraction 5 and fraction 3, in WT and CD9 KO cells, respectively. For quantifications, the mean and standard deviations, from the three Western blots performed are represented. *p<0.05, **p<0.01, ***p<0.001, ****p<0.0001 (for comparison between WT and CD9 KO cells)

SUPPLEMENTARY FIGURE S3

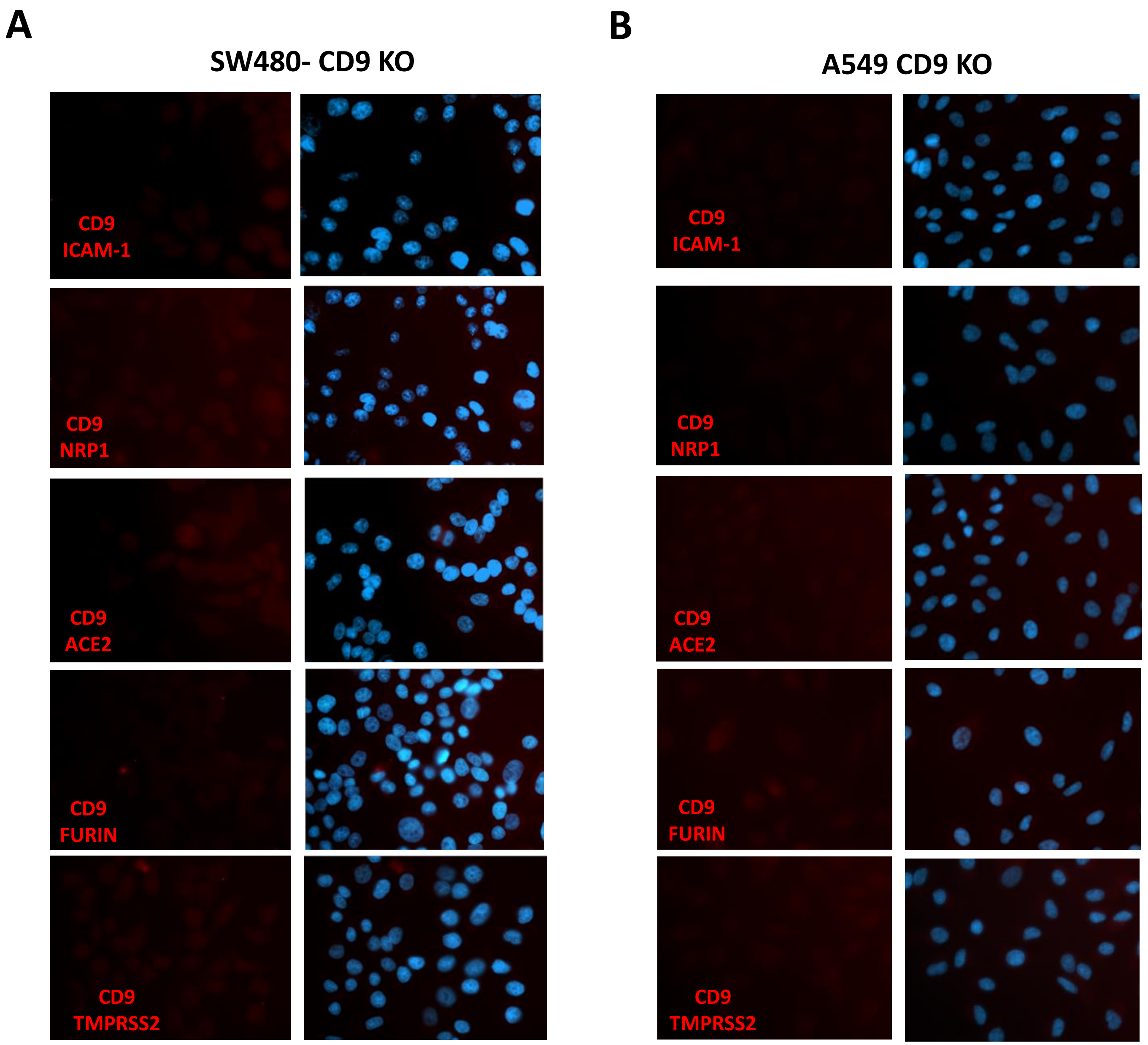

Supplementary Figure S3. Negative controls for PLA experiments confirm assay specificity. Representative images of proximity ligation assay (PLA) performed in CD9 knockout (CD9KO) SW480 (left) and A549-ACE2 (right) cells, showing the absence of PLA signal (red) for all tested interactions. No red fluorescent puncta were detected when probing for CD9 interactions with ICAM-1, NRP1, ACE2, FURIN, or TMPRSS2, confirming the specificity of the PLA signal observed in wild-type cells. Nuclei are stained with DAPI (blue) in all images. Images were acquired at 40× magnification, and 5-8 images were acquired, with each image containing between 80 and 120 cells. The absence of PLA signal in CD9 KO cells validates that the signals detected in wild-type cells depend on the presence of CD9 and are not due to nonspecific antibody binding or background amplification.

SUPPLEMENTARY FIGURE S4

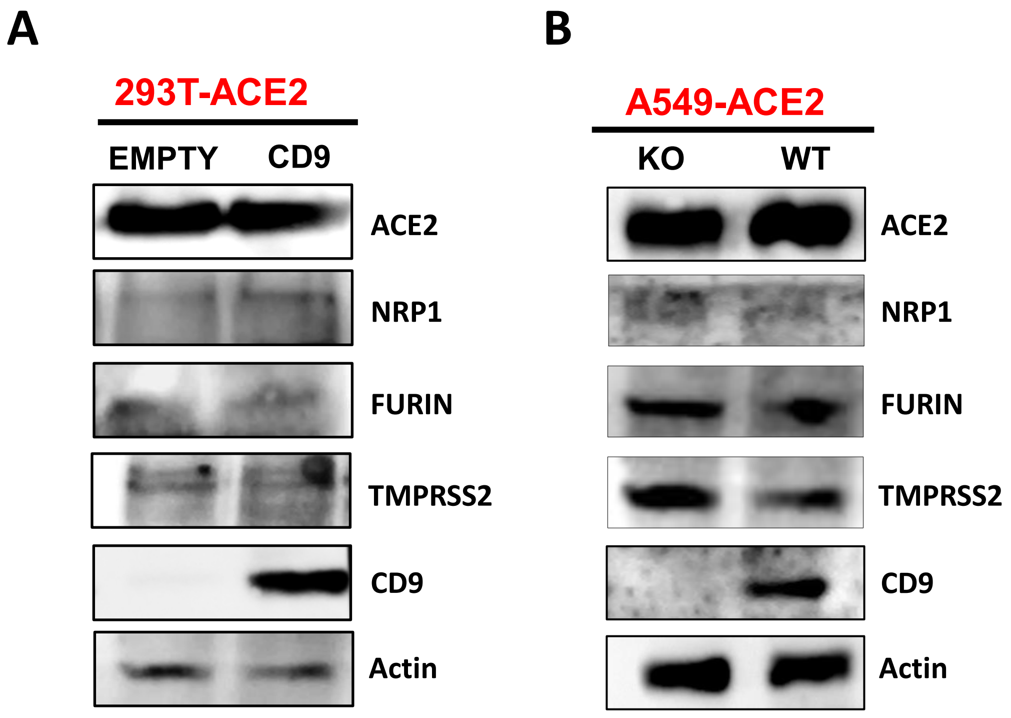

Supplementary Figure S4. ACE2, NRP1, furin, TMPRSS2, CD9 and actin expression in cellular extracts Human 293T cells were transiently co-transfected with a pCAGGS plasmid encoding CD9, or with an empty plasmid (A). Alternatively, A549-ACE2 WT and CD9 KO cells were used (B). **(A and B)** The expression of ACE2, NRP1, furin, TMPRS2, CD9, and actin (as control), was analyzed by Western blot in the cellular extracts.
